# Positional distribution of transcription factor binding sites in *Arabidopsis thaliana*

**DOI:** 10.1038/srep25164

**Published:** 2016-04-27

**Authors:** Chun-Ping Yu, Jinn-Jy Lin, Wen-Hsiung Li

**Affiliations:** 1 Biotechnology Center, National Chung-Hsing University, Taichung 40227, Taiwan; 2 Biodiversity Research Center, Academia Sinica, Taipei 115, Taiwan; 3 Bioinformatics Program, Taiwan International Graduate Program, Institute of Information Science, Academia Sinica, Taipei 115, Taiwan; 4 Institute of Molecular and Cellular Biology, National Tsing Hua University, Hsinchu 300, Taiwan; 5 Department of Ecology and Evolution, University of Chicago, Chicago 60637, USA

## Abstract

Binding of a transcription factor (TF) to its DNA binding sites (TFBSs) is a critical step to initiate the transcription of its target genes. It is therefore interesting to know where the TFBSs of a gene are likely to locate in the promoter region. Here we studied the positional distribution of TFBSs in *Arabidopsis thaliana*, for which many known TFBSs are now available. We developed a method to identify the locations of TFBSs in the promoter sequences of genes in *A. thaliana*. We found that the distribution is nearly bell-shaped with a peak at 50 base pairs (bp) upstream of the transcription start site (TSS) and 86% of the TFBSs are in the region from −1,000 bp to +200 bp with respect to the TSS. Our distribution was supported by chromatin immunoprecipitation sequencing and microarray data and DNase I hypersensitive site sequencing data. When TF families were considered separately, differences in positional preference were observed between TF families. Our study of the positional distribution of TFBSs seems to be the first in a plant.

Binding of a transcription factor (TF) to its DNA binding sites is a critical step to initiate the transcription of its target genes. Typically, a TF binding site (TFBS) is 5 to 15 base pairs (bp) long within the promoter of its target gene and a TF protein usually can recognize a set of similar DNA sequences with varying degrees of binding affinity. In view of the importance of TFBSs in gene regulation, it is useful to know how the TFBSs of a gene are spatially distributed in its promoter region. Such a study for a large number of genes may shed light on how many TFBSs on average regulate a gene and where to find the TFBSs of a new TF.

The spatial distribution of TFBS has been studied in yeast and human^1^,^2^, revealing that TFBSs are not uniformly distributed over the promoter region but tend to lie in the vicinity of the transcription start site (TSS) of the gene. In *Saccharomyces cerevisiae* TFBSs are enriched in the region from 200 bp to 100 bp upstream (from −200 bp to −100 bp) of the transcription start site (TSS) and has a sharp peak at 115 bp^2^. In human, according to the ChIP-chip data of nine TFs, the distribution of TFBSs is a mixture of two distributions: one is a bell-shaped distribution with a narrow peak within 300 bp upstream of the TSS and the other is a uniform background distribution^2^. In plants, TFBSs are enriched in the 200 bp region upstream of the TSS for the stress-responsive genes in *Arabidopsis thaliana*^3^. However, as the study was limited in scale, we conducted a more extensive study.

To study the spatial distribution of TFBSs in an organism requires a large number of known TFBSs in that organism. In plants such data are available only for *Arabidopsis thaliana*. We therefore studied the distribution in this plant. Another requirement for studying the spatial distribution of TFBS is to have a large number of target genes. For this purpose we developed a method for predicting the target genes of a TF whose TFBS is known.

A common experimental approach to identify the TFBSs of a TF in a genome is chromatin immunoprecipitation-sequencing (ChIP-seq) assay^4^. It utilizes a TF-specific antibody to capture *in vivo* cross-linked DNA-protein complexes, which contain the TF protein, DNA fragments, and other chromatin-associated proteins. The DNA fragments are then sequenced to determine the TFBSs (peaks) of the TF over the genome. Many ChIP-seq studies have been made on *A. thaliana*^5^,^6^,^7^. An alternative assay is sequencing of DNase I hypersensitive sites (DHS-seq)^8^,^9^, which detects open chromatin regions that are sensitive to DNase I enzyme to infer the regulatory regions in a genome. In this study, we also used these two types of data to study the positional distribution of TFBSs in *A. thaliana* and we compared the distributions obtained from the three types of data.

## Results

### Statistics of TFBSs in the promoters of predicted target genes of a TF

We collected 586 TFBSs for 400 Arabidopsis TFs from the TF databases and literature (see Materials and Methods). As in Yu *et al.*^10^, for a predicted TFBS in the promoter of a putative target gene of a TF to be considered a target site of the TF it has to pass the conservation test in the following three species: *Arabidopsis lyrata*, *Brassica oleracea*, and *Brassica rapa*. Among the annotated 33,602 genes in *A. thaliana* (TAIR10), 17,573 genes were found to have orthologs in all of the above three species. We found that 547 of the 586 TFBSs passed the conservation test and that 15,781 of the 17,573 genes contained at least one of the 547 TFBSs in their promoter region (Supplementary Table S1), which is defined as the region from 2,000 bp (−2,000 bp) upstream of the transcription start site (TSS) to 200 bp (+200 bp) downstream of the TSS of the gene. On average, a gene has 5.4 TFBSs, which belong to 4.1 TF families, and a TFBS is, on average, present in 162.4 of the 15,781 genes.

### Positional distribution of TFBSs

**Figure 1:**
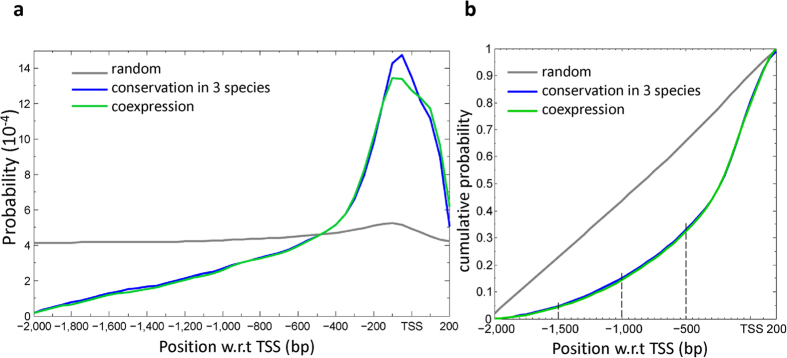
Positional distribution of TFBSs in the promoter of genes in *A. thaliana*. (**a**) Three distributions derived from three different sets of conditions. The gray line shows the distribution of the candidate TFBSs predicted by FIMO in the region from −2,000 to +200 bp with respect to TSS. The blue line is the distribution of the predicted TFBSs that have passed the conservation test in *A. lyrata*, *Brassica oleracea*, and *Brassica rapa*. The green line is the distribution of the TFBSs that have passed both the conservation test and the test of TF-target gene co-expression. Each distribution was normalized such that the integration of the probability over the region was equal to one. (**b**) The cumulative distributions for the three distributions in (**a)**. The three dashed lines indicate the cumulative probabilities up to the site at −1,500 bp, −1,000 bp, or −500 bp from TSS, respectively.

First, we consider the occurrence of a TFBS sequence in the promoter region of a gene. The presence of a TFBS sequence at a nucleotide site in a promoter was predicted by FIMO^11^ (p-value < 10^−4^). Note that this occurrence refers to the chance occurrence of the sequence without requiring that it passes the conservation test. This distribution can be considered the random distribution of TFBS sequences in the promoter region. As expected, this distribution (the probability density) is rather flat except for a small peak at −50 bp with respect to the TSS (the grey line in Fig. 1a; see also Supplementary Fig. S1). The probability density at the peak (5.2 × 10^−4^) is only 1.3-fold higher than that at the position −2,000 bp (~4.1 × 10^−4^).

Second, we consider the positional distribution of the TFBS sequences that have passed the conservation test. We call this the positional distribution of TFBSs. Compared to the random distribution (the grey line in Fig. 1a), this distribution is much more similar to a bell-shaped distribution with a peak at −50 bp (the blue line in Fig. 1a). This distribution shows that the majority of the TFBSs (63%) occur within the region from −400 bp to +200 bp (kurtosis = 0.36). (Kurtosis is a measure of whether the distribution is more peaked or flat compared to the normal distribution, which has kurtosis = 0.) Moreover, the tail of the distribution in the upstream direction drops quickly and the probability density becomes negligibly small beyond (upstream of) −2,000 bp (Supplementary Fig. S2). In comparison, the distribution drops at a much slower rate on the downstream side of the TSS. However, the downstream region of the TSS may include the 5′ UTR region and part of the coding region, which are likely functional, so that a TFBS similar sequence in the downstream region of the TSS may have a higher probability to pass the conservation test than a sequence in an upstream region of the TSS. Note that at +200 bp the distribution is already close to that of the random distribution. At any rate, it should be kept in mind that the inferred distribution in the downstream side of the TSS is likely higher than the actual distribution. Under the distribution given by the blue line in Fig. 1a, the cumulative probability of TFBSs is 4.4% at −1,500 bp (Fig. 1b). This denotes the probability that a TFBS would occur in the region from −2,000 bp to −1,500 bp, or in other words, it is the probability that a TFBS would be missed if the promoter region is defined from −1,500 bp to +200 bp with respect to the TSS. Under the same distribution the accumulative probability is 14.3% at −1,000 bp, and 31.8% at −500 bp (Fig. 1b). We also estimated that the accumulative probability of having a TFBS upstream of −2,000 bp is only 0.0004 (Supplementary Fig. 2).

Third, we add the condition that a TFBS is the target of a specific TF, only if the expression profile of the gene is correlated with that of the TF gene, that is, the Pearson Correlation Coefficient (PCC) is >0.8 (see Materials and Methods). The distribution under this additional condition, which is denoted by the green line in Fig. 1a, is very similar to the blue line. Thus, adding this condition does not change the distribution much. From the above analyses, we conclude that the blue line in Fig. 1a can be taken as the distribution of TFBSs in *Arabidopsis thaliana*.

**Figure 2:**
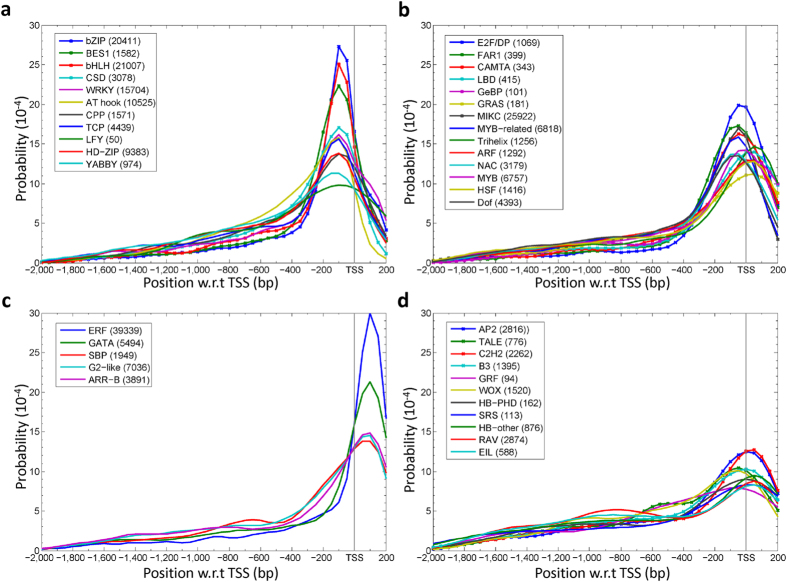
Positional distribution of TFBS in a TF family. A total of 41 distributions are shown and categorized by their peaks and the kurtoses. (**a**) Positional distributions of TFBSs in each of 11 TF families with the distribution peak located at <−100 bp w.r.t TSS and a kurtosis >0, which means a sharper distribution than the normal distribution, which has kurtosis = 0. (**b**) Positional distributions of TFBSs in each of 14 TF families with a distribution peak within the region from −100 bp to TSS and a kurtosis >0. (**c**) Positional distributions of TFBSs in each of 5 TF families with the distribution peak within the region from TSS to +200 bp and a kurtosis >0. (**d**) Positional distributions of TFBSs for each of 11 TF families with a kurtosis <0. For each TF family, the total number of TFBS occurrences is indicated in the parentheses. The vertical line in each diagram represents the position of the TSS.

When the distribution is given for each TF family separately, some distributions turn into sharply bell-shaped, showing a peak upstream of the TSS (Fig. 2a), very close to the TSS (Fig. 2b) or downstream of the TSS (Fig. 2c). For example, the positional preference of ERF (kurtosis = 2.9) and that of GATA (kurtosis = 1.2) (Fig. 2c) are in the region from +100 bp to +200 bp, those of E2F/DP and CAMTA (kurtosis >2.5) (Fig. 2b) are in the region from −100 bp to +100 bp and those of bZIP, bHLH, and BES1 (Fig. 2a) are in the region from −100 bp to −50 bp (kurtosis > 2.1). On the other hand, for a number of TF families, including AP2, TALE and C2H2, the distribution becomes flatter (kurtosis < 0) (Fig. 2d). Thus, different TF families appear to have different positional preferences.

### Positions of DH and ChIP sites

**Figure 3:**
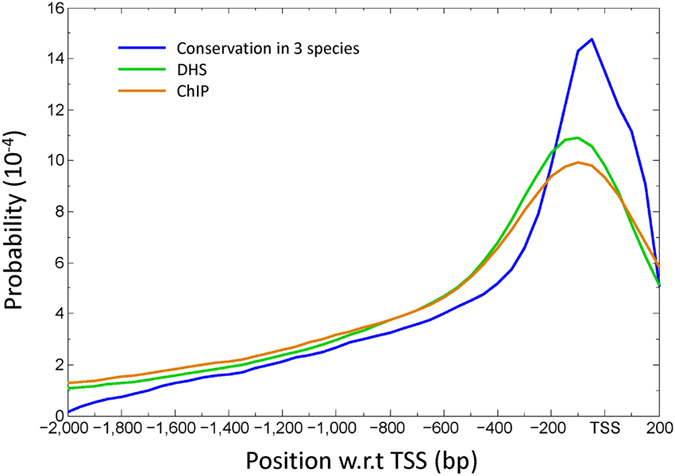
Positions of putative TFBSs associated with inferred DH and ChIP sites. Two distributions of the distances of DH (green) and ChIP (orange) sites from the TSSs of nearest genes are shown. For comparison, the distribution of TFBSs that passed the conservation in three species is also shown (blue).

One may also get some insight into the positional preference of TFBSs from the positional distributions of DNase I hypersensitive sites (DHSs)^12^,^13^ or chromatin immunoprecipitation (ChIP) experiments^14^. For the DH sites we collected from the literature, the data contained ~62,000 peaks with an average site length of 311.7 bp and with a length summation of 19.2 Mbp (16.1% of the genome size) (see Materials and Methods). For the ChIP-seq or ChIP-chip peaks (denoted ChIP for both ChIP-seq and ChIP-chip henceforth), the data included ~56,000 peaks with an average length of 327.4 bp. Since the lengths of peaks in the two datasets have several hundred base pairs, the distance was calculated by the mid position of a DH or ChIP site to the TSS of its nearest gene. The positional distributions of DH and ChIP sites (the green and orange lines in Fig. 3) are similar to that of TFBSs (the blue line in Fig. 3), with peaks near −100 bp. This observation largely supports the positional distribution of TFBSs we obtained above. However, the peaks for DH and ChIP sites are lower than that for TFBSs, perhaps for two reasons. First, the peak for a DH or ChIP site gives only an approximate position for the TFBS—the longer the peak, the less precise the position of the TFBS. Second, the DH and ChIP sites were not required to pass the conservation test, while the TFBSs have passed the conservation test. Note also that the peaks for DH and ChIP sites are located slightly more upstream than the TFBS peak, perhaps partly because, as mentioned above, the conservation test was more favorable for TFBS sequences located downstream of TSS.

**Figure 4:**
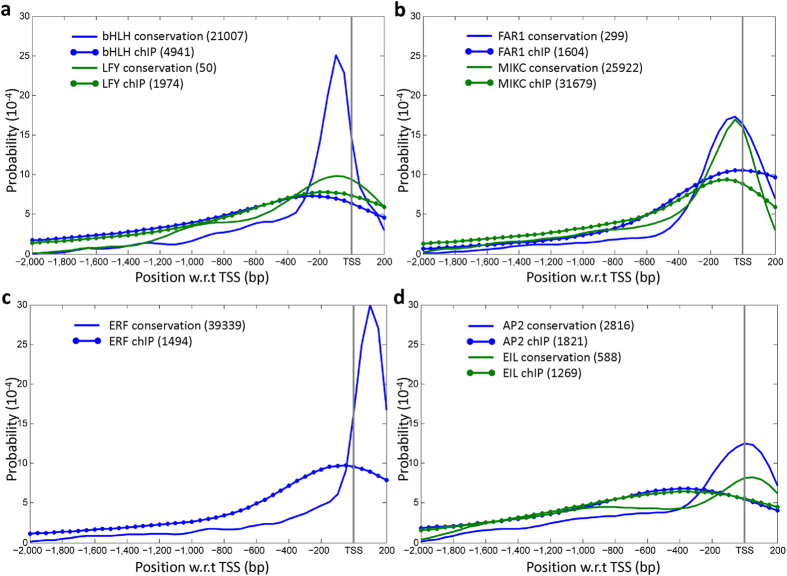
Positional distribution of ChIP sites in a TF family. The distributions of ChIP sites (lines with dots) are shown for 7 different TF families. The corresponding distributions of conserved TFBSs (lines) are also shown for the comparison. The arrangements of TF families in each diagram are the same as in Fig. 2. The position of the TSS is indicated by the vertical line and the numbers of ChIP sites and TFBSs are shown in the parentheses.

As the ChIP dataset provided 27 TFs in 13 TF families, we grouped the ChIP sites into families. As it requires a fairly large number of sites to obtain a reliable distribution, we selected the 7 TF families that had >1000 sites to examine positional preferences between different TF families. Overall, the positional distributions of the ChIP sites in a family have a flatter shape compared to the positional distributions of TFBS (Fig. 4), because the average length of ChIP sites (~300 bp) is considerably longer than that of the TFBS. The peaks of ChIP site distributions tend to locate more upstream than that of the counterpart of the TFBSs, perhaps because the longer ChIP sites make it more difficult to determine the precise TFBS position and because the ChIP sites had not been subjected to the conservation test. The positional distributions of ChIP sites for individual TFs indicate that the TFBSs for different TF families have different positional preferences.

## Discussion

We used ~500 known TFBSs in *A. thaliana* to study the positional distribution within the promoter sequences of ~15,800 genes. The distribution inferred (kurtosis = 0.36) is somewhat sharper than the normal distribution (kurtosis = 0). It has a peak at 50 bp upstream of the TSS and the majority of TFBSs (86%) lie in the region from −1,000 bp to +200 bp with respect to the TSS. When the 41 TF families were considered separately, different positional preferences were found between families and 11TF families showed a flatter distribution (kurtosis < 0) than the normal distribution. However, even for the most flat distribution 79% of the TFBSs are in the region from −1,000 bp to +200 bp. Thus, it is a good region to look for the TFBS(s) of a TF in *A. thaliana*.

In this study, we only used the TFs whose TFBSs have been verified by experiment, so most of the TFBS sequences (518/586 = 88%) were assigned to unique TFs. Two TFBS sequences that are assigned to two TFs in the same TF family usually have substantial differences. Nevertheless, two TFs in the same family can share a target gene for two reasons. First, two sequences on the promoter of the gene are predicted to be similar to the two TFBSs, respectively. This is the same as the situation where two TFs in different TF families share a target gene. In this case, there are two TF binding sites. Second, a single sequence on the promoter of the gene is predicted to be similar to both TFBSs, according to the prediction method such as FIMO, which we used. This can happen because a TF usually recognizes a set of similar sequences and all prediction methods allow for such sequence variation. In this case, we count only one TF binding site.

Our distribution of TFBS in Arabidopsis and the published distributions in yeast and human all show positional preference of TFBSs with respect to the TSS and all have a probability peak upstream of the TSS. However, the probability for a TFBS to lie downstream of the TSS is negligible in yeast but substantial in Arabidopsis and human. As mentioned above, the method used to infer the distribution in Arabidopsis may produce a bias for the region downstream of TSS. For human the distribution was based on ChIP-chip data and as mentioned above this type of data might also have a bias for the downstream region of TSS. Therefore, the distributions in Arabidopsis and human should be reexamined when more suitable data becomes available.

For the human study^2^, the authors proposed that the distribution consists of a narrow peak and a uniform distribution in all 9 TFs studied, especially the homeobox (HB) TFs, and they hypothesized that a TF switches its TFBS between the proximal and the distal region. In our study, there are five TF families that possess the homeodomain (HB or HD), and the distributions for the HB-PHD, HB-other, TALE, and WOX families showed a low peak and long tail (Fig. 2d), although the HD-ZIP family showed a sharp peak and short tail (Fig. 2a), suggesting that some homeodomain TFs in Arabidopsis have a dual binding distribution like some TFs in human.

As the amount of TFBS data used in this study is still limited and as there are still TF families in *A. thaliana* for which no data about the TF binding specificity is available, a more detailed study should be conducted in the future to check the accuracy of the present inferences and to expand the study to other TFs.

## Materials and Methods

### Collection of TF-TFBS pairs

For Arabidopsis TFBSs, we collected 478 TFBSs for 359 TFs from four databases (TRANSFAC, JASPAR, Athamap and CIS-BP^15^,^16^,^17^,^18^) and also 108 TFBSs for 63 TFs from Franco-Zorrilla *et al.*^19^. These two collections provided a total of 586 non-redundant TFBSs for 400 TFs that have been experimentally verified (Supplementary Table 1), using PBM (protein binding array), SELEX (systematic evolution of ligands by exponential enrichment), ChIP-seq, or ChIP-chip.

### TFBS conservation test

For each of the collected TFBSs, we examined whether a TFBS similar sequence is present in the promoter sequence of a gene, using the software FIMO^11^ (p-value < 10^−4^). The promoter sequence of a gene is defined by the region from −2,000 bp to +200 bp relative to the TSS of the gene. Then, we used the following three species to test the conservation of a TFBS in *Arabidopsis thaliana* (TAIR10): *Arabidopsis lyrata* (v.1.0), *Brassica oleracea* (v2.1) and *B. rapa* (IVFCAASv1). The orthologous relationships between *A. thaliana* and the three reference species were obtained from the Ensembl Plants orthologous definition and we determined a one-to-one relationship by two criteria: (i) the sequence identity between the target and the query is >50% and (ii) among the potential orthologs it has the highest average sequence identity with the Arabidopsis gene. If no such ortholog was found for one of the three species, we discarded the gene from our analysis. For a gene under study, the promoter sequences in the three reference species were identified by alignment to the promoter sequence of the orthologous gene in *A. thaliana*. We required that a TFBS similar sequence is located within 100 bp from the TFBS of *Arabidopsis thaliana* in the promoter sequence alignment of the all four species and is on the same strand^10^. Supplementary Fig. 3 shows that in the alignment of promoter sequences of *A. thaliana* and the three other species, 79% of the TFBSs have identical positions in the alignment and in less than 10% of the cases the TFBS position has moved more than 10 bp.

### Target gene prediction

To assess whether a TFBS is regulated by its putative cognate TF, we looked for transcriptome (RNA-seq) data. For Arabidopsis transcriptomes, we collected three data sets: 5 transcriptomes from stomatal development^20^, 6 transcriptomes from developing flower^21^, and 10 transcriptomes from the apical meristem of flow initiation^22^. In total, we collected 21 transcriptomes.

In the 21 transcriptomes, a gene was defined as expressed and retained in subsequent analyses, if its raw RPKM value was >1 in at least two transcriptomes. Under this criterion, there were 32,799 expressed genes in the 21 transcriptomes. For each transcriptome, the gene expression levels were then normalized by upper quantile normalization^23^. A predicted target gene of a TF is said to be co-expressed with the TF gene, if the Pearson Correlation Coefficient (PCC) between their expression levels in the 21 transcriptomes is >0.8.

### DHS and ChIP-seq datasets

Two datasets of DNase I hypersensitive sites (DHSs) were collected. First, a processed dataset of DHS peaks was downloaded from Zhang *et al.*^13^, who used two-week old leaf tissues and closed flower buds of *A. thaliana*. This dataset included 55,500 peaks (FDR < 0.01) with an average length of 311.7 bp. The second set was from the DHS peaks in roots of 7-day-old seedlings of *A. thaliana*^12^. This set provided a total of 43,500 peaks with an average length of 188.8 bp. In these two datasets, two peaks were merged if they overlapped, resulting in a total of 62,300 peaks and a length summation of 19.2 Mbp (Supplementary Table S2).

For the chromatin immunoprecipitation (ChIP) data in *A. thaliana*, we downloaded integrated data of binding profiles for 27 TFs^14^, which used techniques of microarray (ChIP-chip) or deep sequencing (ChIP-seq). It contained a total of 56,600 peaks with an average length of 327.4 bp (Supplementary Table S3).

For the positional distributions of DHS and ChIP-seq peaks, we calculated the distance between the mid position of the peak and the TSS of its nearest gene in either the forward or the reverse strand.

## Additional Information

**How to cite this article**: Yu, C.-P. *et al.* Positional distribution of transcription factor binding sites in Arabidopsis thaliana. *Sci. Rep.* **6**, 25164; doi: 10.1038/srep25164 (2016).

## Supplementary Material

Supplementary Information

Dataset 1
