# Supplementary material for: Positional distribution of transcription factor binding sites in Arabidopsis thaliana
Source: Sci Rep. 2016 Apr 27;6:25164. doi: 10.1038/srep25164 (PMC4846880; doi:10.1038/srep25164)
Supplement: Supplementary Information [file srep25164-s1.pdf]

Supplementary Figures

Positional distribution of transcription factor binding sites in *Arabidopsis thaliana*

Chun-Ping Yu[1], Jinn-Jy Lin[2,3,4], and Wen-Hsiung Li[1,2,5,]*

[1]Biotechnology Center, National Chung-Hsing University, Taichung, Taiwan 40227
[2]Biodiversity Research Center, Academia Sinica, Taipei, Taiwan 115
[3]Bioinformatics Program, Taiwan International Graduate Program, Institute of Information Science, Academia Sinica, Taipei, Taiwan 115
[4]Institute of Molecular and Cellular Biology, National Tsing Hua University, Hsinchu, Taiwan 300
[5]Department of Ecology and Evolution, University of Chicago, Chicago 60637, USA

*Corresponding author: Email: whli@sinica.edu.tw

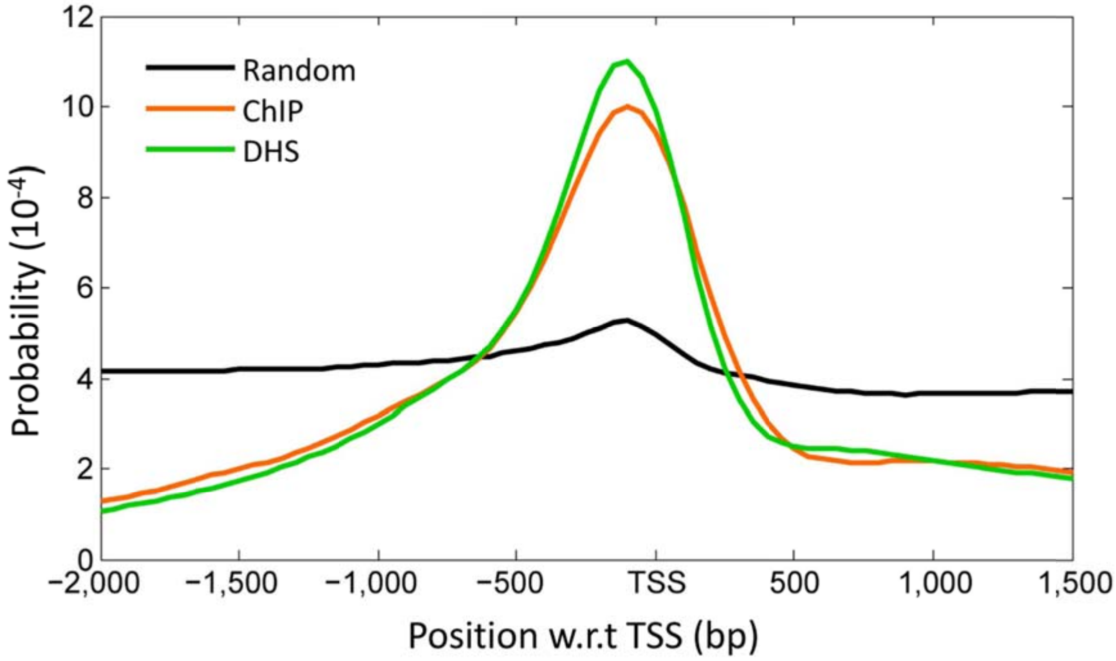

Figure S1. Positional distributions of random TFBSs, DH and ChIP sites. Random TFBSs refer to the TFBSs predicted by FIMO (p-value$<10^{-4}$) in the region from -2,000 bp to +1,500 bp with respect to the TSS of the gene. The random distribution of TFBSs is represented by the black line. The orange and green lines show the distributions of the distances from ChIP and DH sites to the TSS of their nearest genes, respectively. For comparison, the three distributions were scaled such that the integration of the probabilities over the region from -2,000 bp to +200 bp w.r.t the TSS was one.

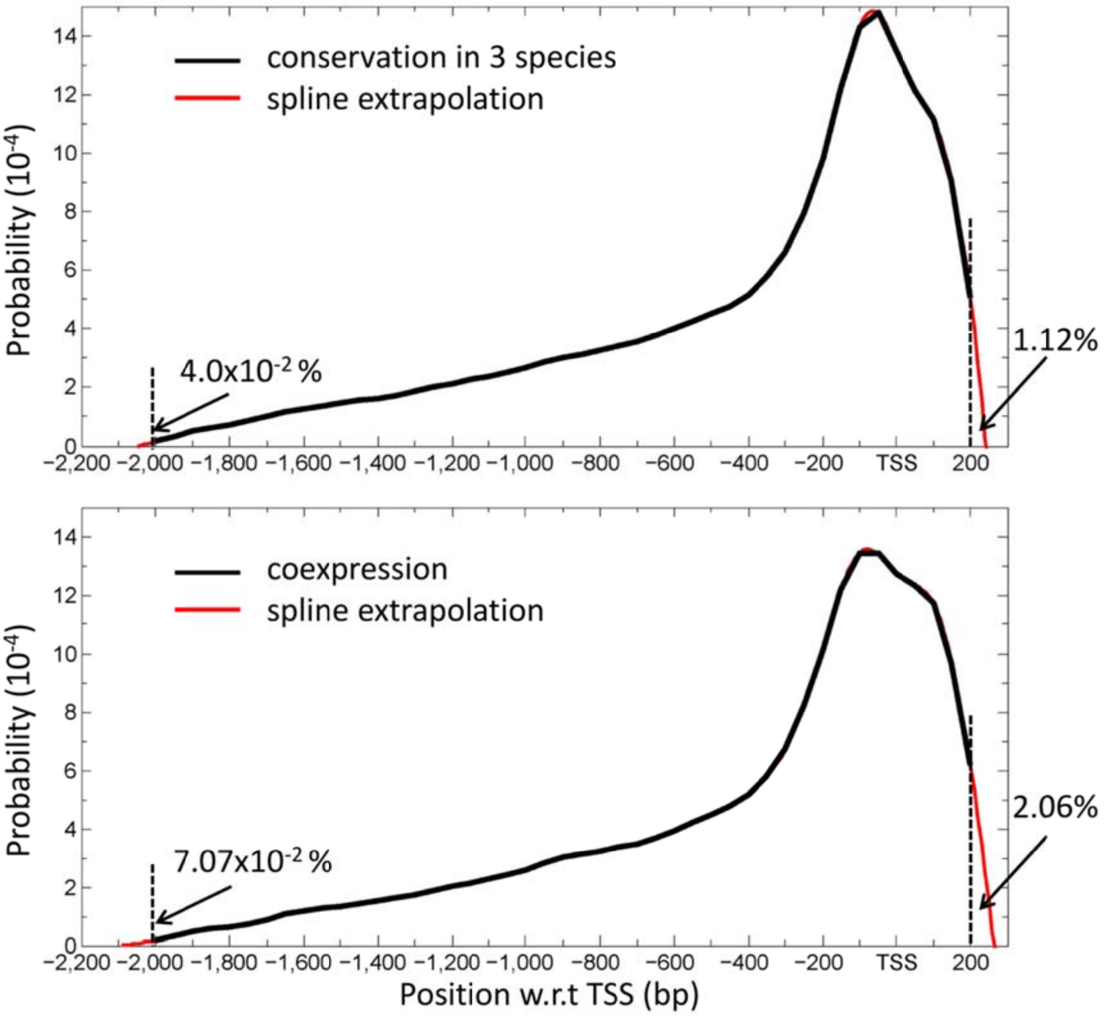

Figure S2. Extrapolation of the positional distributions of TFBSs. The positional distributions of putative TFBSs (black lines) under the conservation criterion and the extra criterion of coexpression are shown in the upper and the lower diagram, respectively. The distributions are extended from both ends of the region from -2,000 bp to +200 bp w.r.t the TSS untill the sites that the probability density reached zero (red), using the cubic spline extrapolation. The cumulative probabilities outside the region are indicated.

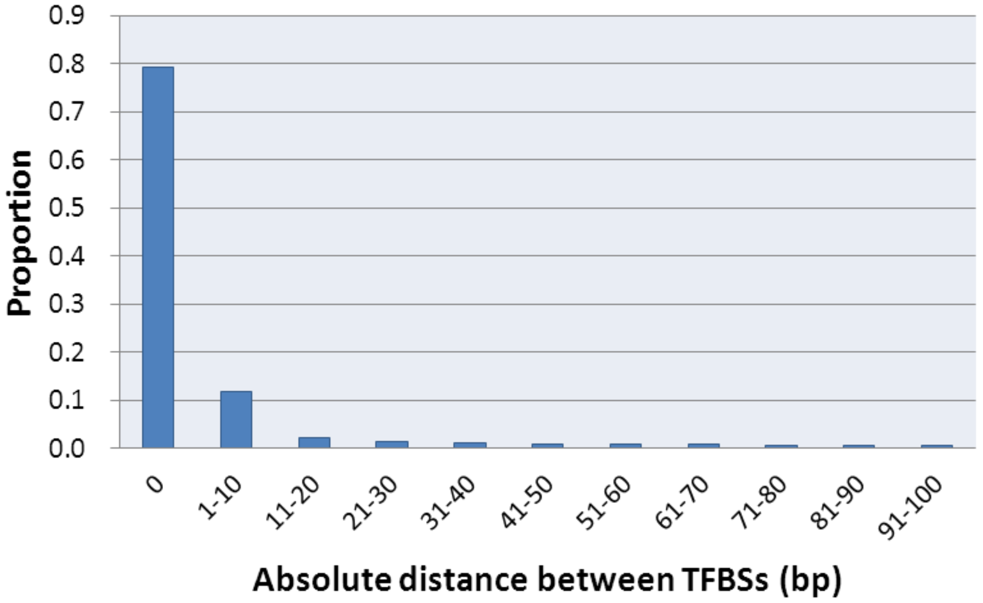

Figure S3. Distribution of the distances between the genomic positions of TFBSs in *A. thaliana* and in the reference species. The x-axis indicates the absolute distance (base pairs) between the TFBS in *A. thaliana* and that in a reference species in the alignment of the promoter sequences (including gaps) of the four species. The y-axis is the proportion of the number of predicted target genes in a bin among the total number of predicted target genes.
